# Views of Rural US Adults About Health and Economic Concerns

**DOI:** 10.1001/jamanetworkopen.2019.18745

**Published:** 2020-01-08

**Authors:** Mary G. Findling, Robert J. Blendon, John M. Benson, Justin M. Sayde, Carolyn E. Miller

**Affiliations:** 1 Department of Health Policy and Management, Harvard T.H. Chan School of Public Health, Boston, Massachusetts; 2 Research, Evaluation, and Learning Unit, Robert Wood Johnson Foundation, Princeton, New Jersey

## Abstract

**Importance:**

The 2016 US presidential election brought national visibility to prominent economic issues affecting rural America, yet little has been done to examine serious health or economic problems in rural communities from the perspectives of rural US adults themselves.

**Objective:**

To examine views on the most serious health and economic concerns and potential solutions in rural communities.

**Design, Setting, and Participants:**

This survey study used 2 cross-sectional probability-based telephone (cellular and landline) surveys of adults living in the rural United States. The first survey was conducted June 6 to August 4, 2018, and the second survey was conducted January 31 to March 2, 2019. All results were weighted to be nationally representative of rural US adults. Data analysis was conducted in April 2019.

**Main Outcomes and Measures:**

The primary study outcomes were the prevalence of rural adults reporting their biggest community problems, problems with health care access and costs, ratings of the local economy, views on opioid addiction, and views on solving major community issues.

**Results:**

The 2018 survey included 1300 respondents (response rate, 12%; completion rate, 70%), and the 2019 survey included 1405 respondents (response rate, 8%; completion rate, 76%). Rural adults primarily identified as non-Hispanic white (78%), and more than half of respondents were 50 years or older (survey 1: 55%, survey 2: 53%). Rural adults identified opioid or other drug addiction or abuse (25%; 95% CI, 22%-28%) and economic concerns (21%; 95% CI, 19%-24%) as the most serious problems facing their local communities. Opioid or other drug addiction or abuse was reported as a serious problem in their local community by 57% (95% CI, 53%-60%) of rural adults, while 49% (95% CI, 46%-53%) of rural adults said they personally know someone who has had opioid addiction. Problems paying medical bills were reported by 32% (95% CI, 29%-36%) of rural adults, and recent issues with accessing health care were reported by 26% (95% CI, 23%-30%) of rural adults. Regarding financial and economic issues, 55% (95% CI, 52%-59%) of rural adults rated their local economy as only fair or poor, and 49% (95% CI, 44%-54%) of rural adults said they would have difficulty paying off an unexpected $1000 expense. On solving major community problems, 58% (95% CI, 54%-62%) of rural adults saw a need for outside help to solve major community problems, and among those who reported needing outside help, 61% (95% CI, 57%-66%) identified a major role for government.

**Conclusions and Relevance:**

These findings suggest that in today’s economically stretched rural United States, opioid or other drug addiction or abuse has emerged as an equal problem with economic concerns. One in 3 rural adults still have problems paying their medical bills even after the passage and implementation of the Patient Protection and Affordable Care Act. Although rural communities have traditionally been self-reliant, more than half of rural adults are open to outside help to solve serious problems facing their communities, including major help from government.

## Key Points

**Question:**

What are rural US adults’ views on serious health and economic concerns facing their communities?

**Findings:**

This survey study of 1300 adults in 2018 and 1405 adults in 2019 found that opioid or other drug addiction or abuse and economic concerns were identified as the most serious problems facing rural communities. Rural adults also reported recent problems with paying medical bills and accessing health care.

**Meaning:**

These findings suggest that in today’s economically stretched rural United States, opioid or other drug addiction or abuse has emerged as a significant problem on the same level with economic concerns.

## Teaser

This survey study uses nationally representative surveys from 2018 and 2019 to examine the perspectives of adults in the rural United States on the biggest issues facing their communities, including health care and the economy.

## Introduction

The 2016 US presidential election brought national visibility to prominent economic issues affecting rural communities, including persistent poverty, limited job growth, and a slow economic recovery from the Great Recession of 2007 to 2009.^1,2,3^ Although economic issues and national security have historically been identified by the public as the most serious problems facing the nation,^4^ US adults often have different views about serious problems facing their local communities. To our knowledge, no prior studies have recently addressed US rural public opinion on serious health and economic issues facing their communities, including opioid addiction, health care costs, and the government’s role in solving major problems.

In particular, rural perspectives on the recent opioid epidemic remain understudied. In 2016, more than 1.5 million people living in rural areas misused opioids, including prescription pain relievers and heroin, and nearly 5000 people in rural areas died of opioid overdose—the largest annual opioid-related death toll in US history.^5^ Rates of drug overdose deaths in rural areas (17.0 deaths per 100 000 people) have now surpassed those in urban areas (16.2 deaths per 100 000 people),^6^ President Donald Trump has identified the opioid abuse epidemic as a public health emergency, and the Department of Health and Human Services recently awarded more than $1 billion in grants to combat the opioid crisis.^7^

In this survey study, we conducted 2 original polls in 2018 and 2019. Our objective was to examine the views of rural US adults on serious health and economic problems facing their communities.

## Methods

### Study Design and Sample

Data were obtained from 2 original, nationally representative, probability-based telephone (cellular telephone and landline) polls of rural US adults. Rural was defined as living in areas that are not part of a metropolitan statistical area. The first poll was conducted June 6 to August 4, 2018, and the second poll was conducted January 31 to March 2, 2019. Both surveys were jointly designed by the Harvard T.H. Chan School of Public Health, the Robert Wood Johnson Foundation, and National Public Radio, while SSRS, an independent research firm, administered the survey. The study was determined to be not human subjects research by the Harvard T.H. Chan School of Public Health Office of Human Research Administration and was therefore exempt from review because researchers were not directly involved in data collection and deidentified data sets were used for analysis.

The samples for these surveys included 1300 adults in 2018 and 1405 adults in 2019 aged 18 years and older. The response rate for survey 1 was 12% and the response rate for survey 2 was 8%, calculated based on the American Association for Public Opinion Research’s RR3 formula.^8^ Among respondents who answered initial demographic screening questions, the completion rates were 70% for survey 1 and 76% for survey 2. While these response rates are relatively low, they are within the typical range of response rates for telephone polling by prominent survey organizations.^9^ Several studies have shown that such surveys, when based on probability samples and weighted using US Census parameters, yield accurate estimates in most cases compared with objective measures and with higher-response surveys.^9,10,11,12^ For instance, a recent study showed that across 14 different demographic and personal characteristics, the mean difference between government estimates from high-response rate surveys and a Pew Research Center poll with a response rate of 9%, which is similar to rates for our surveys, was 3 percentage points.^9^ These measures included employment status, household size, health insurance status, length of residence at current address, marital and parenthood status, smoking, and having a driver’s license. Because data from our polls were drawn from a probability sample and used the best sampling and weighting practices available in current polling methods (eg, making live telephone calls rather than automated calls, using >50% cellular telephone samples, and calling back nonrespondents up to 6 times at varying points in the day), they were expected to provide accurate results consistent with surveys with higher response rates. These samples were reliably generalizable to the broader population of adults living in the rural United States, within a margin of error of 3.6 percentage points at the 95% CI.

### Survey Instrument

Both polls asked about rural adults’ views on serious health and economic problems facing themselves and their communities, with some questions only asked among a randomized half sample to reduce respondent burden. We analyzed 21 questions from the surveys. Full question wording is available in the eAppendix in the Supplement. We examined respondents’ views on the overall biggest problem facing their local communities, biggest problem facing their families, and most urgent health problem facing their local communities. Respondents reported recent problems with health care access, costs, and quality, hospital closures, and telehealth use. Respondents rated the seriousness of several public health problems facing rural communities (ie, opioid or other drug addiction or abuse, suicide, and homelessness) and indicated whether they personally knew someone, such as a friend or family member, who has had opioid addiction. Respondents rated the strength of their local economy and reported any difficulty paying for unexpected expenses and medical or dental treatment.

We examined perspectives on solving community problems by asking about respondents’ confidence that major problems facing the local community would be solved in the next 5 years and views on whether communities could solve major problems mostly on their own or would need outside help. Among adults who reported their community would need outside help, we asked which of 6 groups (or something else) they believed would play the greatest role in solving their community’s major problems: state government, federal government, county or regional government, big businesses, nonprofit organizations (including charities), religious organizations, or something else.

We collected demographic information from respondents to better understand differences by rural region. Mutually exclusive groups were defined as rural Appalachia, following the Appalachian Regional Commission definition,^13^ and non-Appalachian rural Northeast, South, Midwest, and West, following US Census definitions.^14^

### Statistical Analysis

After calculating descriptive statistics, we analyzed the prevalence of adults who reported their views or experiences for each question. We also conducted a subgroup analysis on the overall biggest problem facing respondents’ local communities by region because of heterogeneity among regional rural populations.^15^ Two-tailed *t* tests were used to assess statistical differences among 5 rural regions, and we only report results with at least 10 percentage points’ difference as robust enough to have practical implications. For all analyses, statistical significance was determined at *P* < .05.

To compensate for known biases in telephone polling (eg, nonresponse bias) and variations in probability of selection within and across households, sample data were weighted by household size and composition, cellular telephone and landline use, and demographic characteristics (ie, sex, age, education, race/ethnicity, and region using US Census data) to reflect the true population distribution of adults in the rural United States. Other techniques, including random-digit dialing, replicate subsamples, and random selection of a respondent within a household, were used to ensure that the sample is representative. All analyses were conducted using Stata statistical software version 15.0 (StataCorp), and all tests accounted for the variance introduced by weighted data. Data analysis was conducted in April 2019.

## Results

The samples for these surveys included 1300 adults in survey 1 in 2018 and 1405 adults in survey 2 in 2019. The characteristics of rural adults included in this study are presented in Table 1. Rural adults primarily identified as non-Hispanic white (78%), while 8% identified as Hispanic or Latino, and 8% as non-Hispanic black. Eighty-one percent of rural adults did not have a college degree. More than half of respondents were 50 years or older (survey 1: 55%, survey 2: 53%), while 44% of respondents in survey 1 and 47% of respondents in survey 2 were aged 18 to 49 years.

**Table 1.** Characteristics of Nationally Representative Samples of Rural US Adults

Characteristic	Weighted %^a^	
	2018 Survey 1 (n = 1300)	2019 Survey 2 (n = 1405)
Race/ethnicity		
Non-Hispanic white	78	78
Hispanic or Latino	8	8
Non-Hispanic black	8	8
Other	6	7
Sex		
Women	50	49
Men	50	51
Age, y		
18-29	18	19
30-49	26	28
50-64	30	28
≥65	25	25
Education		
No college degree^b^	81	81
College degree or more	19	19
Household annual income, $		
<25 000	31	34
25 000 to <50 000	23	24
50 000 to <75 000	16	17
≥75 000	21	25
Rural region^c^		
Appalachia	19	18
Northeast	6	6
Midwest	30	30
South	31	31
West	15	15

^a^ Weighted percentages of adults were estimated with survey weights to adjust for unequal probability of sampling. Percentages may not total 100% owing to rounding and responses of don’t know or refused that are included in the total sample size but not reported here.

^b^ Includes those with some college experience (including business, technical, or vocational school after high school) but no college degree, as well as those with a high school degree, general educational development certificate, or less.

^c^ Regions were mutually exclusive. Rural Appalachia was defined by county using the Appalachian Regional Commission definition. All other regions were defined using the US Census.

Table 2 presents rural adults’ views on serious community health and economic problems. When asked to identify the overall biggest problem facing their local community as an open-ended question, 25% (95% CI, 22%-28%) of rural adults identified opioid or other drug addiction or abuse, and 21% (95% CI, 19%-24%) of rural adults reported economic concerns, including availability of jobs, poverty, businesses closing, cost of living, and low wages. Notably, adults living in rural Appalachia were significantly more likely to rate opioid or other drug addiction or abuse as the biggest problem facing their community (42%; 95% CI, 34%-51%) compared with all other regions (Northeast: 19%; 95% CI, 10%-35%; *P* = .003; Midwest: 19%; 95% CI, 15%-25%; *P* < .001; South: 19%; 95% CI, 14%-25%; *P* < .001; West: 28%; 95% CI, 20%-37%; *P* = .02). When asked about the most urgent health*-*specific problem currently facing their local community as an open-ended question, 23% (95% CI, 20%-26%) of rural adults identified opioid or other drug addiction or abuse, 12% (95% CI, 10%-14%) of rural adults identified cancer, and 11% (95% CI, 9%-13%) of rural adults identified access to health care. When asked about the biggest problem facing themselves and their families as an open-ended question, rural adults most commonly cited financial problems (27%; 95% CI, 24%-30%), then health or health care problems (16%; 95% CI, 14%-19%), or no problems (14%; 95% CI, 12%-17%). Opioid addiction was reported as a serious problem in their local community by 57% (95% CI, 53%-60%) of rural adults, including 33% (95% CI, 29%-36%) saying it was a very serious problem, while 49% (95% CI, 46%-53%) of rural adults said they personally know someone who has had opioid addiction. Homelessness was reported as a problem in their local community by 33% (95% CI, 29%-38%) of rural adults, including 15% (95% CI, 12%-19%) of rural adults who said it was a major problem. Suicide was reported as a serious problem by 31% (95% CI, 27%-34%) of rural adults, including 12% (95% CI, 10%-15%) of rural adults who said it was a very serious problem. Local hospital closures during the past few years were reported by 8% (95% CI, 6%-12%) of rural adults.

**Table 2.** Rural US Adults’ Perspectives on Major Health and Economic Concerns

Response	Sample Size, No.^a^	Weighted % (95% CI)^a^
**2018 Survey 1**		
Overall biggest problem facing your local community^b^^,^^c^^,^^d^	1300	
Opioid or other drug addiction or abuse		25 (22-28)
Economy or jobs		21 (19-24)
Most urgent health problem currently facing your local community^b^^,^^c^^,^^d^	1300	
Opioid or other drug addiction or abuse		23 (20-26)
Cancer		12 (10-14)
Access to health care		11 (9-13)
Overall biggest problem facing you and your family^c^^,^^d^	1300	
Financial problems		27 (24-30)
Health or health care problems		16 (14-19)
None		14 (12-17)
Rating your local economy	1300	
Excellent		8 (6-11)
Good		36 (33-40)
Only fair		34 (31-38)
Poor		21 (18-24)
Rating opioid addiction as a problem in your local community	1300	
Very serious		33 (29-36)
Somewhat serious		24 (21-27)
Not too serious		4 (3-6)
Not a problem		31 (28-34)
Personally know someone who has had opioid addiction	1300	
Yes		49 (46-53)
No		50 (47-54)
Rating suicide as a problem in your local community	1300	
Very serious		12 (10-15)
Somewhat serious		19 (16-22)
Not too serious		4 (3-6)
Not a problem		60 (57-64)
**2019 Survey 2**		
Difficulty paying family medical bills or for dental treatment	1405	
Major problems		19 (17-22)
Minor problems		13 (11-16)
No problems		67 (64-70)
Would have a problem paying off an unexpected $1000 expense right away^e^	691	
Yes		49 (44-54)
No		50 (45-55)
Recently could not get access health care when needed	1405	
Got health care every time it was needed		72 (69-75)
Needed health care but did not get it		26 (23-30)
Reasons you could not get health care you needed (among those who needed health care but did not get it)	331	
Could not afford that health care		45 (38-52)
Felt the health care location was too far or too difficult to get to		23 (18-30)
Could not get an appointment during the hours you needed		22 (17-29)
Could not find a physician who would take your health insurance		19 (14-26)
Experienced recent problems with health care quality you received in the past few years^e^	701	
Yes		28 (24-33)
No		70 (65-75)
Closure of any local hospital in the past few years^e^	704	
Yes		8 (6-12)
No		90 (87-93)
Rating homelessness as a major problem in your local community^e^	714	
Major problem		15 (12-19)
Minor problem		18 (14-22)
Not a problem		62 (57-66)

^a^ Unweighted sample size and weighted percentages are reported. Percentages were estimated with survey weights to adjust for unequal probability of sampling. Percentages may not total 100% owing to rounding and responses of don’t know or refused that are included in the total sample but not reported here.

^b^ Opioid or other drug addiction or abuse includes drug addiction or abuse (general, drug type unspecified, or did not specifically mention opioids) and specific mentions of opioid addiction or abuse (including heroin and prescription pain relievers). Economic concerns include the availability of jobs, poverty, businesses closing, cost of living, and low wages.

^c^ Administered as an open-ended question; top mentions reported.

^d^ No other issues were mentioned by more than 10% of rural adults.

^e^ To reduce respondent burden, this question was only asked to a randomized half sample.

Regarding health care, 32% (95% CI, 29%-36%) of rural adults said their family has experienced problems affording medical bills or dental treatment in the past few years, including 19% (95% CI, 17%-22%) of rural adults who reported major problems. Among adults with lower incomes (those with household incomes <$25 000 per year), 44% (95%, CI, 38%-51%) cited problems affording medical bills or dental treatment. For health care access, 26% (95% CI, 23%-30%) of rural adults said there has been a time in the past few years when they needed health care but did not get it, including 24% (95% CI, 21%-27%) of rural adults with health insurance. When those who did not receive care when needed were given a list of potential reasons why they did not get care, 45% (95% CI, 38%-52%) of rural adults said they could not afford that care, while 23% (95% CI, 18%-30%) of rural adults cited problems with inaccessible health care locations, 22% (95% CI, 17%-29%) of rural adults reported difficulty getting appointments, and 19% (95% CI, 14%-26%) of rural adults reported they could not find a physician who would accept their health insurance. On health care quality, 28% (95% CI, 24%-33%) of rural adults said they felt there was a problem with the quality of health care they had received in the past few years.

On economic issues, 55% (95% CI, 52%-59%) of rural adults held negative views on their local economy, including 34% (95% CI, 31%-38%) of rural adults rating it as only fair and 21% (95% CI, 18%-24%) of rural adults rating it as poor. Forty-nine percent (95% CI, 44%-54%) of rural adults said they would have a problem immediately paying off an unexpected $1000 expense.

Table 3 presents rural adults’ views about solving serious community problems. Rural adults were split in their optimism that major problems facing their local community would be solved in the next 5 years, with 51% (95% CI, 47%-54%) saying they were confident (including 12% [95% CI, 10%-15%] who were very confident) and 46% (95% CI, 42%-50%) reporting they were not confident (including 18% [95% CI, 15%-21%] who were not at all confident). Regarding solving major community problems, 58% (95% CI, 54%-62%) of rural adults reported believing their community would need outside help, while 37% (95% CI, 34%-41%) of rural adults reported believing their community could solve these problems mostly on its own. Among rural adults who believed their community would need outside help, 61% (95% CI, 57%-66%) identified government as playing the greatest role in solving community problems, including their state government (30%; 95% CI, 26%-35%), federal government (18%; 95% CI, 15%-22%), and their county or regional government (13%; 95% CI, 10%-17%). An additional 13% (95% CI, 10%-17%) of rural adults said big businesses would play the greatest role in solving community problems, while 6% (95% CI, 4%-9%) of rural adults identified nonprofit organizations, 5% (95% CI, 3%-7%) of rural adults said religious organizations, and 7% (95% CI, 5%-10%) of rural adults said something else.

**Table 3.** Perspectives of Rural US Adults on Solving Major Community Health Problems

Response	Sample Size, No.^a^	Weighted % (95% CI)^a^
Confidence that major problems facing your local community will be solved in the next five years	1300	
Very confident		12 (10-15)
Somewhat confident		38 (35-42)
Not too confident		28 (25-31)
Not at all confident		18 (15-21)
Views on solving community problems on its own or with outside help	1290	
Can accomplish mostly on its own		37 (34-41)
Will need outside help		58 (54-62)
Views on which groups will play the greatest role in solving major community problems (among those saying community will need outside help)	755	
Your state government		30 (26-35)
The federal government		18 (15-22)
Your county or regional government		13 (10-17)
Big businesses		13 (10-17)
Nonprofit organizations, including charities		6 (4-9)
Religious organizations		5 (3-7)
Something else		7 (5-10)

^a^ Unweighted sample size and weighted percentages are reported. Percentages were estimated with survey weights to adjust for unequal probability of sampling. Percentages may not total 100% owing to rounding and responses of don’t know or refused that are included in the total sample but not reported here.

Rural adults’ experiences with telehealth are shown in Table 4. Twenty-four percent (95% CI, 21%-27%) of rural adults said they had received a health care diagnosis or treatment over the past few years via email, text messaging, live text chat, a mobile application, live video, or telephone, including 15% (95% CI, 13%-18%) using a telephone specifically and 14% (95% CI, 12%-16%) using other electronic means (ie, email, text, live chat, mobile application, or video). Rural adults who used telehealth used it for a variety of purposes; more than half of rural adults (53%; 95% CI, 46%-60%) said they had obtained a prescription using telehealth, while fewer rural adults reported having used telehealth for a chronic condition (25%; 95% CI, 19%-32%), an emergency (16%; 95% CI, 11%-23%), or an infectious disease (9%; 95% CI, 6%-14%). When adults who used telehealth were asked about reasons for telehealth use, 69% (95% CI, 62%-76%) said they used telehealth because it was the most convenient way to get a diagnosis or treatment.

**Table 4.** Experiences of Rural US Adults With Telehealth

Telehealth Use	Sample Size, No.^a^	Weighted % (95% CI)^a^
Recently received health care diagnosis or treatment via	1405	
Email, text messaging, live text chat, mobile application, live video, or telephone		24 (21-27)
Email, text messaging, live text chat, mobile application, or live video		14 (12-16)
Telephone		15 (13-18)
Reasons for telehealth use among rural telehealth patients	360	
Most convenient way to get diagnosis or treatment		69 (62-76)
Could not see regular physician in person		30 (24-37)
Too difficult to travel to physician or hospital		26 (20-33)
Medical purpose of telehealth use among telehealth patients	360	
For a prescription from physician or other health professional		53 (46-60)
For diagnosis or treatment for a chronic condition		25 (19-32)
For diagnosis or treatment for an emergency		16 (11-23)
For diagnosis or treatment for an infectious disease		9 (6-14)

^a^ Unweighted sample size and weighted percentages are reported. Percentages were estimated with survey weights to adjust for unequal probability of sampling. Percentages may not total 100% owing to rounding and responses of don’t know or refused that are included in the total sample but not reported here.

## Discussion

This survey study examines rural US adults’ views on serious health and economic problems facing their communities today, with 4 principal findings. First, while another survey^16^ fielded concurrently with this study showed dissatisfaction with government, immigration, and economic issues as the top concerns of the public nationally, we found that opioid or other drug addiction or abuse has emerged as an equally serious problem as economic issues among rural US adults, especially among adults living in rural Appalachia. Consistent with other recent national surveys,^17^ we found that rural adults expressed serious concerns about opioid abuse as a major threat to their local communities, corresponding to a rapidly evolving epidemic with a historic death toll in rural areas.^5,18,19^ These levels of public concern are potentially important for influencing the views of policy makers about appropriate actions and spending.^20^ During a period of significant new government action on treating and preventing opioid abuse, our results suggest there is wide public support for redirecting policy makers’ attention and funds toward handling the opioid epidemic in rural areas. However, additional research is needed on the attitudes of rural adults toward prevention, policy, and treatment options, as well as related stigma.

Second, despite the passage and implementation of the Patient Protect and Affordable Care Act^21^ during the past decade, we found that one-fourth of rural adults still reported problems with health care costs and access. Inability to pay medical bills and problems with access to health care remain significant burdens for rural adults, and while telehealth use is increasing, a low percentage of the population currently uses it to manage chronic diseases or emergencies. Our results are consistent with other research findings suggesting that the rural population has limited health care options,^22^ and US populations across the urban-suburban-rural continuum continue to experience issues with health care costs, access, and quality.^23^

Third, despite a strong national economy,^24^ more than half of rural adults rated their local economy as fair or poor, and about half of rural adults reported they would have difficulty paying an unexpected $1000 expense. These results are troubling indicators of financial insecurity among rural adults, as poverty rates have remained higher in rural areas compared with nonrural areas for decades.^15^ Alongside a slow economic recovery from the 2007 to 2009 recession and voting patterns documented in the 2016 presidential election,^15,25^ these results show rural populations continue to struggle economically. What has been added to these serious problems is the emergence of the opioid epidemic.

Fourth, we found that more than half of rural US adults identified a need for outside help to solve major local problems, including seeing a major role for government specifically. These findings run counter to other narratives highlighting the self-reliant nature of rural communities, where rural adults have been found to frequently oppose government involvement in community problems.^26,27,28^

In addition, we found that there was less public concern about suicide than about opioid or other drug addiction or abuse. This finding is important because increasing suicide rates have gained national attention, with people living in rural areas significantly more likely to die by suicide than people in urban areas.^29^ Trends in public concern over these issues should be tracked through time to monitor awareness and public support for interventions.

### Limitations

Our results should be interpreted considering several limitations. Self-reporting, question wording, ordering, and the amount of questions asked limited our ability to fully assess rural adults’ perspectives and experiences. This includes the inability of these surveys to separately examine cost, access, and quality differences by type of health care (eg, primary, specialty, emergency, dental, or mental health care). Nonresponse bias is also a concern in public opinion surveys, although evidence suggests that low response rates do not bias results if the survey sample is representative of the study population.^9,10^ Recent research has shown that such surveys, when based on probability samples and weighted using US Census parameters, yield accurate estimates in most cases compared with both objective measures and higher-response surveys.^9,10,11,12^ However, it is still possible that some nonresponse bias may remain that is related to the experiences or views being measured. In addition, asking about sensitive topics, such as suicide and opioid addiction, may have biased rural adults to underreport or overreport concerns, depending on their personal experiences and awareness of these issues as public health problems. Despite these limitations, this study allowed us to examine the views of rural adults on serious community problems among 2 large national samples of adults living in the rural United States.

## Conclusions

This survey study found that in the current economically stretched rural United States, opioid or other drug addiction or abuse has emerged as an equal problem with economic concerns, and approximately half of rural adults personally know someone who has had opioid addiction. In addition, one-third of rural adults still have problems paying their medical bills even after the passage and implementation of the Patient Protection and Affordable Care Act. Although rural communities have traditionally been self-reliant, more than half of rural adults are open to outside help in solving serious problems facing their communities, including significant help from government.

## Notes

Supplement. **eAppendix.** Surveys 1 and 2

## References

[1] US Department of Agriculture, Economic Research Service Rural employment and unemployment. https://www.ers.usda.gov/topics/rural-economy-population/employment-education/rural-employment-and-unemployment/. Accessed April 19, 2019.

[2] Miller K, Weber B; Rural Policy Research Institute Report Persistent poverty dynamics: understanding poverty trends over 50 years. http://www.rupri.org/Forms/Poverty_MillerWeber_July2014.pdf. Accessed April 19, 2019.

[3] Goetz SJ, Partridge MD, Stephens HM The economic status of rural America in the President Trump era and beyond. Appl Econ Perspect Policy. 2018;40(1):-. doi:10.1093/aepp/ppx061

[4] Aisch G, Parlapiano A What do you think is the most important problem facing this country today? *New York Times* February 27, 2017. https://www.nytimes.com/interactive/2017/02/27/us/politics/most-important-problem-gallup-polling-question.html. Accessed April 19, 2019.

[5] Center for Behavioral Health Statistics and Quality 2018 Surveillance report of drug-related risks and outcomes. https://www.cdc.gov/drugoverdose/pdf/pubs/2018-cdc-drug-surveillance-report.pdf. Accessed April 19, 2019.

[6] Mack KA, Jones CM, Ballesteros MF Illicit drug use, illicit drug use disorders, and drug overdose deaths in metropolitan and nonmetropolitan areas—United States. MMWR Surveill Summ. 2017;66(19):1-12. doi:10.15585/mmwr.ss6619a1 29049278 PMC5829955

[7] Department of Health and Human Services HHS awards over $1 billion to combat the opioid crisis. https://www.hhs.gov/about/news/2018/09/19/hhs-awards-over-1-billion-combat-opioid-crisis.html. Accessed April 19, 2019.

[8] American Association for Public Opinion Research Standard definitions: final disposition case codes and outcome rates for surveys. https://www.aapor.org/AAPOR_Main/media/publications/Standard-Definitions20169theditionfinal.pdf. Accessed September 24, 2019.

[9] Keeter S, Hatley N, Kennedy C, Lau A; Pew Research Center What low response rates mean for telephone surveys. https://www.pewresearch.org/methods/2017/05/15/what-low-response-rates-mean-for-telephone-surveys/. Accessed April 19, 2019.4

[10] Kohut A, Keeter S, Doherty C, Dimock M, Christian L Assessing the representativeness of public opinion surveys. Pew Research Center. https://www.people-press.org/2012/05/15/assessing-the-representativeness-of-public-opinion-surveys/. Accessed April 19, 2019.

[11] Yaeger DS, Krosnick JA, Chang L, Comparing the accuracy of RDD telephone surveys and internet surveys conducted with probability and non-probability samples. Public Opin Q. 2011;75(4):709-747. doi:10.1093/poq/nfr020

[12] Keeter S, Kennedy C, Dimock M, Best J, Craighill P Gauging the impact of growing nonresponse from a national RDD telephone survey. Public Opin Q. 2006;70(5):759-779. doi:10.1093/poq/nfl035

[13] Appalachian Regional Commission Counties in Appalachia. https://www.arc.gov/appalachian_region/CountiesinAppalachia.asp. Accessed April 19, 2019.

[14] US Census Bureau Census Bureau regions and divisions with state FIPS codes. https://www2.census.gov/geo/pdfs/maps-data/maps/reference/us_regdiv.pdf. Accessed October 23, 2019.

[15] US Department of Agriculture Rural America at a glance: 2017 edition. https://www.ers.usda.gov/webdocs/publications/85740/eib-182.pdf?v=0. Accessed October 25, 2019.

[16] Gallup The most important problem. https://news.gallup.com/poll/1675/most-important-problem.aspx. Accessed April 19, 2019.

[17] Blendon RJ, Benson JM The public and the opioid-abuse epidemic. N Engl J Med. 2018;378(5):407-411. doi:10.1056/NEJMp1714529 29298128

[18] Kertesz SG Turning the tide or riptide: the changing opioid epidemic. Subst Abus. 2017;38(1):3-8. doi:10.1080/08897077.2016.1261070 27858590

[19] Sigmon SC Access to treatment for opioid dependence in rural America: challenges and future directions. JAMA Psychiatry. 2014;71(4):359-360. doi:10.1001/jamapsychiatry.2013.4450 24500040

[20] SteelFisher GK, Blendon RJ, Lasala-Blanco N, Blendon RJ, Lasala-Blanco N Ebola in the United States: public reactions and implications. N Engl J Med. 2015;373(9):789-791. doi:10.1056/NEJMp1506290 26222381

[21] Patient Protection and Affordable Care Act. Pub L No. 111-148. 42 USC (2010).

[22] Douthit N, Kiv S, Dwolatzky T, Biswas S Exposing some important barriers to health care access in the rural USA. Public Health. 2015;129(6):611-620. doi:10.1016/j.puhe.2015.04.001 26025176

[23] Sommers BD, McMurtry CL, Blendon RJ, Benson JM, Sayde JM Beyond health insurance: remaining disparities in US health care in the post-ACA era. Milbank Q. 2017;95(1):43-69. doi:10.1111/1468-0009.12245 28266070 PMC5339398

[24] Crustinger M US economy grew at strong 3.5 percent rate in Q3. *Associated Press* October 26, 2018 https://apnews.com/3baa9cfd9191472fb9f44004131ebeff. Accessed October 25, 2019.

[25] Scala DJ, Johnson KM Political polarization along the rural-urban continuum: the geography of the presidential vote, 2000-2016. Ann Am Acad Pol Soc Sci. 2017;672:162-184. doi:10.1177/0002716217712696

[26] Pew Research Center American trends panel wave 32. https://www.pewsocialtrends.org/dataset/american-trends-panel-wave-32/. Accessed April 19, 2019.

[27] Gimpel JG, Karnes KA The rural side of the urban-rural gap. PS Polit Sci Polit. 2006;39(3):467-472. doi:10.1017/S1049096506060859

[28] Cramer Walsh K Putting inequality in its place: rural consciousness and the power of perspective. Am Polit Sci Rev. 2012;106(3):517-532. doi:10.1017/S0003055412000305

[29] Centers for Disease Control and Prevention Americans in rural areas more likely to die by suicide. https://www.cdc.gov/media/releases/2017/p1005-rural-suicide-rates.html. Accessed April 19, 2019.

