# Supplementary material for: Views of Rural US Adults About Health and Economic Concerns
Source: JAMA Netw Open. 2020 Jan 8;3(1):e1918745. doi: 10.1001/jamanetworkopen.2019.18745 (PMC6991316; doi:10.1001/jamanetworkopen.2019.18745)
Supplement: Supplement. — eAppendix. Surveys 1 and 2 [file jamanetwopen-3-e1918745-s001.pdf]

Supplementary Online Content

Findling MG, Blendon RJ, Benson JM, Sayde JM, Miller CE. Views of rural US adults about health and economic concerns. *JAMA Netw Open*. 2020;3(1):e1918745. doi:10.1001/jamanetworkopen.2019.18745

eAppendix. Surveys 1 and 2

This supplementary material has been provided by the authors to give readers additional information about their work.

© 2020 Findling MG et al. *JAMA Network Open.*

eAppendix. Surveys 1 and 2

S1 – Survey 1, conducted June 6 to August 4, 2018 among a nationally representative sample of 1,300 rural U.S. adults ages 18+

S2 – Survey 2, conducted January 31 to March 2, 2019 among a nationally representative sample of 1,405 rural U.S. adults ages 18+

Questions about the most serious community/individual problems

1. (S1) What would you say is the biggest problem facing your local community? (open-ended)

2. (S1) What is the biggest problem facing you and your family? (open-ended)

3. (S1) What is the most urgent health problem currently facing your local community? (open-ended)

Rating local economy

4. (S1) Overall, how would you rate the economy in the region where you live and work? Would you say it is excellent, good, only fair, or poor?

Opioid addiction

5. (S1) Now I'd like to ask you some questions about opioid addiction. When we ask about opioids, we mean strong painkillers, such as Percocet, OxyContin, Vicodin, or fentanyl.
Is people being addicted to opioids a problem in your local community, or not?

(Asked of those who say opioid addiction is a problem in their local community)
5a. (S1) How serious a problem for your local community is people being addicted to opioids? Is it a very serious problem, somewhat serious, or not too serious?

6. (S1) Do you personally know someone, such as a friend or family member, who has struggled with opioid addiction, or not?

Suicide

7. (S1) Now I'd like to ask you some questions about mental health. Is suicide a problem in your local community, or not?

(Asked of those who think suicide is a problem in local community; n=440)
7a. (S1) How serious a problem is suicide for your local community? A very serious problem, somewhat serious, or not too serious?

Problems with health care costs and paying unexpected expenses

8. (S2) Within the past few years, have you or anyone in your family ever had a problem paying for your medical bills or dental treatment, or not?

8a. (S2) IF YES, ASK: Was that a major problem or a minor problem?

© 2020 Findling MG et al. *JAMA Network Open.*

9. (S2) *(Asked of half-sample C; n=691)* Suppose you had an unexpected expense, and the amount came to one thousand dollars. Based on your current financial situation, would you have a problem paying off the full amount of that expense right away, or not?

Health care access
10. (S2) Was there any time in the past few years when you needed health care but did not get it, or did you get health care every time you needed it in the past few years?

11. (S2) Please tell me whether or not any of the following were reasons you could not get the health care you needed. How about (INSERT ITEM)? Was that a reason you did not get the health care you needed? How about (INSERT ITEM)?

11a. You could not afford that health care
11b. You could not find a doctor who would take your health insurance
11c. You could not get an appointment during the hours you needed
11d. You felt the health care location was too far or difficult to get to

Health care quality
12. (S2) *(Asked of half-sample A; n=701)* Was there any time in the past few years when you felt there was a problem with the quality of health care you received, or have you not had any problems with the quality of health care you received in the past few years?

Hospital closures
13. (S2) *(Asked of half-sample B, n=704)* In the past few years, have any hospitals in your local community closed down?

Homelessness
14. (S2) *(Asked of half-sample D; n=714)* Is homelessness a problem in your local community, or not?

14b. (S2) IF YES, ASK: Would you say that is a major problem or a minor problem?

Solving Local Problems

15. (S1) In terms of the major problems facing your local community, how confident are you, if at all, that these problems will be solved in the next five years? Very confident, somewhat confident, not too confident, or not at all confident?

16. (S1) When it comes to solving these major problems, is this something you think your local community can accomplish mostly on its own, or do you think it will need outside help?

17. (S1) *(Asked of those who say their community will need outside help; n=755)* Which ONE of the following groups do you think will play the greatest role in solving these problems? Your state government; the federal government; your county or regional government; big businesses; nonprofit organizations, including charities; religious organizations; or something else?

© 2020 Findling MG et al. *JAMA Network Open.*

Telehealth Use
18. (S2) In the past few years, have you ever received a diagnosis or treatment from a doctor or other health care professional using email, text messaging, live text chat, a mobile app, or a live video like FaceTime or Skype?

19. (S2) In the past few years, have you ever received a diagnosis or treatment from a doctor or other health care professional over the telephone?

20. (S2) *(Asked of those who ever used telehealth)* Have you ever used [email, text messaging, live text chat, a mobile app, or a live video] OR [the telephone] (INSERT ITEM), or not?

20a. To get a diagnosis or treatment for a chronic condition
20b. To get a diagnosis or treatment for an infectious disease
20c. To get a diagnosis or treatment for an emergency
20d. To get a prescription from your doctor or other health professional

21. (S2) *(Asked of those who ever used telehealth)* Please tell me if each of the following was or was not a reason why you used [email, text messaging, live text chat, a mobile app, or a live video] OR [the telephone] to get a diagnosis or treatment from a doctor or other health care professional. How about (INSERT ITEM)? Was that a reason, or not?

21a. You couldn't see your regular doctor or other health professional in person
21b. It was too hard to travel to the doctor, other health professional, or hospital
21c. It was the most convenient way to get a diagnosis or treatment

© 2020 Findling MG et al. *JAMA Network Open.*
